# Metabolism, microbiome and colorectal cancer

**DOI:** 10.18632/aging.101234

**Published:** 2017-04-28

**Authors:** Somenath Datta, Sanjib Chowdhury, Hemant K. Roy

**Affiliations:** Department of Medicine, Boston University Medical Center, Boston, MA 02118, USA

**Keywords:** gut microbiome, microbial dysbiosis, Warburg effect, colorectal cancer

Alterations in metabolism is one of the newly discovered molecular hallmarks of cancer [1]. The preferential utilization of glycolysis over oxidative phosphorylation (“Warburg Effect”) even in normoxic condition is a common theme in many malignancies including colorectal cancer (CRC). From a teleological perspective, the loss of efficiency of ATP generation is believed to be offset by the production of building blocks necessary for cell growth and proliferation. Our group has recently extended these findings to the premalignant colonic mucosa supporting the role of early metabolic reprogramming to a Warburg-like physiology as one of the initiating factors in colon carcinogenesis [2].

One critical unanswered question is, what are the biological drivers of metabolic alterations in early colon carcinogenesis? An emerging candidate is the gut microbiome which is increasingly recognized as a regulator of both host metabolism and immune response [3]. Intriguingly, dysregulation of the gut microbiome has been linked to a variety of human diseases from malignancies (e.g. CRC) along with metabolic diseases including diabetes and obesity. For colon carcinogenesis, population dynamics (microbial dysbiosis) with decreasing the population of “good microbes” (e.g., *L. acidophilus*, *L. rhamnosus, S. thermophilus*, *F. prausnitzii, A. muciniphila, B. breve* and *B. longum*) and increasing “bad microbes” (e.g., *H. pylori, A. spp, E. faecalis, Genotoxic B. fragilis, Genotoxic E. Coli, F. nucleatum, S. bovis, S. spp* and *C. spp*) can lead to chronic inflammation and oncogenic induction and progression[4]. Although the exact mechanisms of microbial influence on the development of CRC remains to be fully established, the major contributions are balancing between pro- and anti-inflammatory signals, microbial enterotoxins affecting host intracellular pathways, conversion of pro-carcinogenic dietary factors and xenobiotics into carcinogens and microbial fermentation of inaccessible carbohydrates like dietary fibers and resistant starch resulting in short chain fatty acids (SCFAs). The SCFAs, specifically butyrate, have been shown to reduce cellular proliferation and induce apoptosis in both tumor cells and colonic mucosa of neoplasia harboring patients (field carcinogenesis) [5]. Reports show that diets with high fiber and carbohydrates can facilitate the growth of “good microbes” that produce butyrate. It has been reported that SCFAs regulate the energy metabolism of host cells by activating AMP-activated protein kinase (AMPK) pathway which subsequently triggers lipid and glucose metabolism along with mitigating carcinogenesis.

While progress in the field has been rapid, there are many unanswered questions related to the complexity of the microbiome-host interactions. For instance, the host genetic makeup interacts with the microbiome in a manner consonant with the well-established genetic-environmental interactions that is characteristic of colon carcinogenesis. Moreover, the microbiome is dynamic and impacted by diet among a number of other factors. Indeed, the interactions of diet, genetic substrate have been shown to contribute to all aspects of malignant and metabolic diseases. Specifically, the interactions between diet and microbiome have been shown to mediate diabetes and obesity along with cancer risk [6].

We posit that the link between the microbiome and disease risk, especially CRC, may be mediated via altered colonic mucosal metabolism. This might lead to increased proliferation, generation of reactive oxygen species etc. resulting in injured epithelial cells including stem cells. This is particularly apropos since the number of stem cell divisions is intimately associated with CRC risk thus providing a link between cancer prevalence and aging[7]. From a public health perspective, the link between metabolism and CRC risk may provide insights into the observation that the increase CRC rate is markedly increasing in younger (age < 50) patients in contradistinction to the overall population trends. This mirrors epidemiological data on the rising incidence of obesity and diabetes. There is strong data that obesity/diabetes can foster mucosal metabolic abnormalities in glycolytic and lipogenic pathways, altered AMPK and sirtuin function. Given the incontrovertible evidence that diabetes and obesity are significant CRC risk factors, this may provide a mechanism for the dramatically rising CRC rates in young patients. From a clinical perspective, detecting metabolic changes is of paramount importance for risk stratification especially for patients who fall outside the general screening range. This applies to both patients younger and the older population, since current guidelines recommend average risk screening for only patients age 50 to 75. From a therapeutic perspective, there is interest in targeting these metabolic alterations with agents such as metformin as a promising chemopreventive strategy.

In summary, the report that metabolic reprogramming may be an early event in colon carcinogenesis opens vistas of cancer screening and prevention. Moreover, these findings may represent a biological underpinning between the microbiome and CRC and provide a putative mechanism through which diabetes/obesity may increase risk of colon carcinogenesis.
